# Quackery and the Newspaper Press

**Published:** 1852-11

**Authors:** 


*Quackery and the Newspaper Press.*—It is not often that we find instances of the effrontery of quackery receiving its just deserts at the hands of the newspaper press. The following notice, which we cut from the *New York Freeman's Journal*, is therefore a curiosity worth preserving. If editors, generally, would adopt the same upright policy, society would be much the gainer:

"Dr. J. X. Chabert, Professor of Chemistry, &c., sends us a pamphlet just published, containing his 'Observations on the origin and treatment of Cholera,' with certificates of various cures professed to have been performed by a specific of his preparing. In sending this to us, as we are not a Doctor in medicine, Dr. Chabert seems to desire of us an *extra-professional* notice or opinion. This notice, therefore, cannot be on the medical merits of his pamphlet, but on the aspect in which it is presented. We must say, then, that he pretends to cure cholera by a specific or mode of treatment which he keeps a secret; and that doing this is the thing known as *quackery*, and that quackery is an unprofessional, and is considered a not very honest nor reputable way of making money, and seems a violation of the oath taken by every man that obtains the degree of M. D. in any of the medical schools of this country. Being non-medical ourselves, this is all we are competent to say on the subject."

